# Effects of Mobilization Time on Occurrence of New Fractures after Vertebroplasty

**DOI:** 10.1155/2019/9292617

**Published:** 2019-06-03

**Authors:** Ahmet Onur Akpolat, Sinan Karaca

**Affiliations:** ^1^Department of Orthopaedics and Traumatology, Fatih Sultan Mehmet Training and Research Hospital, Istanbul, Turkey; ^2^Department of Orthopaedics and Traumatology, Sancaktepe Training and Research Hospital, Istanbul, Turkey

## Abstract

**Introduction:**

Osteoporotic vertebral fracture treatment options include vertebroplasty, in which development of new fractures is among the possible complications which may develop during the postoperative period. We aim to evaluate whether or not postoperative mobilization time has effect on occurrence of new fractures.

**Materials and Methods:**

A total of 126 patients, consisting of 30 (39.7%) males and 96 (60.3%) females, who underwent sedation-assisted vertebroplasty under local anesthesia between January 2014 and June 2017 were retrospectively evaluated. Preoperative and postoperative visual analogue scores (VASs) and mobilization time (hours) were assessed. Day of new fracture occurrence during follow-up was assessed.

**Results:**

The mean follow-up period was 9 months (7–13 months). The most common fracture segment was the L1 vertebra (15.9%). The preoperative VAS was 8.29 ± 0.95, and the postoperative VAS was 2.33 ± 0.91. The change in VAS was statistically significant (*p*=0.01, *p* < 0.05). Of all the patients, 21 (16.66%) had developed new fractures. No statistical difference was observed between mobilization time (hours) and formation of new fractures (*p*=0.48, *p* > 0.05).

**Conclusion:**

We came to the conclusion that mobilization time (hours) was not a risk factor in the development of new fractures. In addition, there is no relationship between mobilization time and localization of new fractures.

## 1. Introduction

According to current information, osteoporosis is a disease characterized by increased fragility of the bone leading to fractures, low bone density, and deterioration of the microstructure of bone tissue [1, 2]. The most common type is primary osteoporosis which starts between the ages of 40 and 50; the incidence is 75% between ages of 60 and 70 and 85–90% over age 70 [3].

Every year about 700,000 cases of new osteoporotic fractures are reported in the United States and 1.4 million cases in Europe [4, 5]. The vertebra is among the high-risk regions for osteoporotic fractures [4]. About 83% of vertebral fractures are osteoporotic fractures [6].

Among surgical treatments for osteoporotic vertebral fractures, percutaneous surgical procedures including vertebroplasty and kyphoplasty are commonly performed [7, 8]. Several complications may develop during the early- and late-term postoperative period [9–13]. New fractures are among these complications. Various risk factors have been identified to be associated with occurrence of new fractures in the postoperative period [14, 15]. However, it is unclear whether or not mobilization time (hours) is a risk factor.

There is no clear opinion on when patients should be mobilized in the postoperative period. Mobilization time (hours) is decided based on each surgeon's personal experience. Our aim is to investigate whether there is a relationship between the patient's postoperative mobilization time and a new vertebral fracture.

## 2. Materials and Methods

Patients who underwent sedation-assisted vertebroplasty operation under local anesthesia between January 2014 and June 2017 at the Orthopedics and Traumatology Department were retrospectively evaluated. The study's inclusion and exclusion criteria are listed in Table 1. Of the 126 patients that conformed to appropriate criteria, 30 (39.7%) were male and 96 (60.3%) were female. The mean age of the patients was 63.4 (60–76) years. Preoperative visual analogue scores (VASs) of the patients were assessed. A single dose of enoxaparin sodium was administered 12 hours before the operation and 1 gram cefazolin sodium 30 minutes before the operation. After anesthetic preparation, the patient was brought to a prone position. After appropriate region covering, 1.5 cc midazolam was administered intravenously and sedation was achieved. Localizations of the pedicles were identified with fluoroscopy, and 2% prilocaine was applied to soft tissue surrounding procedure areas. The bone was approached with a 0.5 cm incision. An 11 G vertebroplasty needle of 150 mm in length (SOMATEX® Medical Technologies, Teltow, Germany) was advanced towards the upper outer end of the pedicle. The vertebroplasty needle was advanced to the front third portion of the corpus. After attaining adequate position, cement (V-READY G21) was prepared. Cement of proper consistency was carefully injected with 2 mL canules under fluoroscopic guidance. The operation was terminated if there was the slightest leakage outside of the corpus or strong resistance against the injection. If no problem was encountered, the injection continued until it reached the posterior third boundary of the corpus. A mean amount of 3.6 cc (3.0–4.4) cement was used. As the cement changed due to room temperature, the maximum curing time was 30 minutes [16]. Postoperative VASs of the patients were evaluated at certain intervals. At the VAS of 3 or less, after maximum curing time and wearing off of the sedation effect, the patients were mobilized. Mobilization times (hours) of the patients were noted. Day of postoperative occurrence of new fractures was assessed during follow-up.

## 3. Statistical Evaluation

The IBM SPSS Statistics 22.0 package (IBM SPSS, Turkey) was used for statistical analysis of data attained from the study. A *p* value less than 0.05 was considered statistically significant.

In the analysis of the data, mean, standard deviation, frequency, and percentage values were used to present descriptive statistics.

In the evaluation of the difference between the measurements of the fracture and mobilization time in the new fracture and adjacent segment, the *t*-test was used for independent sampling. In the analysis of the difference between the VAS and the pretest, the *t*-test was used for dependent sampling.

## 4. Results

**Figure 1:**
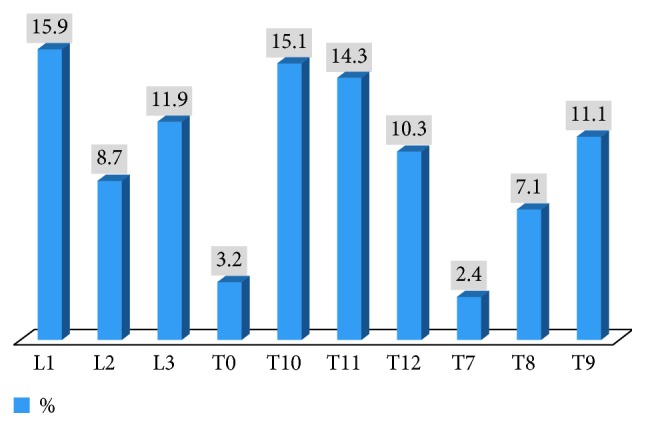
Fracture levels and incidence rates of patients who underwent vertebroplasty procedure.

The mean follow-up length of the patients who participated in the study was 9 (7–13) months. The most common fracture segment was the L1 vertebra (15.9%). In Figure 1, each fracture level and percentages of incidence are shown in detail.

**Figure 2:**
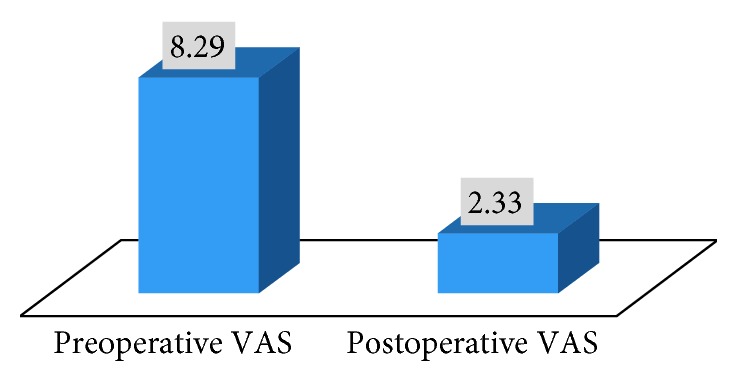
Change in VASs in the preoperative and postoperative period.

While the preoperative VAS was 8.29 ± 0.95, the early-term postoperative VAS was 2.33 ± 0.91. This sudden change in VAS was considered statistically significant (*p*=0.01, *p* < 0.05) (Table 2 and Figure 2).

A total of 21 (16.66%) of the patients showed new fractures during follow-up. The relationship between mobilization time (hours) and new fractures was evaluated. No statistically significant difference was observed between patients who developed and those who did not develop new fractures (*p*=0.75, *p* > 0.05) (Table 3).

Eleven (8.73%) of the patients had new fractures in the adjacent segment and 10 (7.93%) in nonadjacent segments. There was no statistically significant difference in terms of mobilization time (hours) and new adjacent and nonadjacent fracture development (*p*=0.48, *p* > 0.05) (Table 4).

## 5. Discussion

We observed that mobilization time (hours) was not an effective risk factor for new fractures during the early- and midterm postoperative period following vertebroplasty. In addition, there was no effective role in adjacent and nonadjacent segment localization in patients with new fractures. Improvement in VASs after vertebroplasty was prominent. This also confirms how effective the operation is in eliminating pain.

This sudden change in VAS is frequent in the literature. A review by Lenke et al. reported a prominent improvement in pain using preoperative and postoperative scoring systems (VAS, SF-36, and Oswestry) before and after vertebroplasty and kyphoplasty procedures [17]. McGraw et al. reported a notable improvement in VASs after vertebroplasty surgery [18]. The cause of this sudden change in VAS is still unclear. There are several theories regarding this matter. Early studies believed this was due to damage to nerve endings from thermal necrosis or chemical lysis, while recent studies suggest that the pain is mechanical in origin and cement injection prevents periosteal and interosseous nerve tension [19–21].

New fractures are one of the complications of vertebroplasty. There are several studies on whether the new fractures are caused by the procedure or another new independent fracture. A meta-analysis by Zhang et al. did not find a significant difference between conservative treatment, vertebroplasty, and kyphoplasty in terms of new fracture development [22]. However, Trout et al. reported that vertebroplasty increased the risk of new fractures [23]. There is no clear opinion in the literature on the relationship between vertebral fracture treatments and new fractures. General view and findings support that formation of new fractures is based on many different factors. These independent factors include bone mineral density, presence of osteonecrosis in other vertebral bodies, restoration rate of fractured vertebra, history of fractures, intradiscal cement leakage, and distribution of cement filling [14]. The literature reports that intradiscal cement leakage is an especially important risk factor for new fractures [24, 25]. Although intradiscal cement leakage, which plays the largest role in new fracture development, and osteonecrosis of other vertebral bodies were excluded from the study, we found a 16.66% incidence rate of new fractures.

Some of the severe complications which may occur during the early-term postoperative period following vertebroplasty surgery were also noted. These include intradural cement leakage [26], epidural vein cement leakage following spinal stenosis [27], paraplegia [9], pulmonary embolism severe enough to cause mortality [10, 11], paradoxical cerebral embolism [12], and osteomyelitis [13]. Similar clinical symptoms were not observed in the patients of our study.

In the literature, there is not yet a study that evaluates whether or not there is a relationship between postoperative mobilization time and new fractures. For this reason, a comparison with the literature could not be made.

Our study had limitations. These include our study being retrospective in nature and that some of the independent factors (cement filling and restoration rate) were excluded. Furthermore, our study did not evaluate the relationship between localization of new vertebral fractures and onset time. The reason for this is that there are several studies in the literature on this subject and our conviction that these data obtained from our study would have no additional contribution to the literature.

In conclusion, early mobilization of patients undergoing an effective procedure such as sedation-assisted vertebroplasty performed under local anesthesia has no effect on the risk of new fractures. We hope our study will shed light upon potential prospective randomized blind studies on this subject.

## Figures and Tables

**Table 1:** Inclusion and exclusion criteria of the study.

Inclusion criteria	Exclusion criteria
Detection of compression fracture in spinal radiography (minimum 15% loss of height)	Severe cardiopulmonary comorbidity
Spinal fracture at or below Th5	Systemic or local spinal infection
Back pain despite 6-week conservative treatment	Suspicion of underlying malignancy
≥5 visual analogue score (VAS)	Radicular syndrome, spinal cord compression syndrome
Bone edema in MRI of vertebral fracture	Patients exempt from MRI
Focal sensitivity at fracture level during physical examination	Senile dementia (check clinical results) or other cerebral diseases, untreated therapeutic anticoagulation
≤−2.5 bone mineral density	Bone metabolism disease
No intradiscal cement leakage	Allergy against radio-opaque agents
	History of vertebral fracture
	Presence of osteonecrosis in other vertebral bodies

**Table 2:** Change in preoperative and postoperative VASs was statistically significant.

VAS	*n*	*X*	Statistical significance	*p*
Preoperative	126	8.29	0.95	0.01
Postoperative	126	2.33	0.91	

**Table 3:** Statistical results of evaluation of the relationship between mobilization time (hours) and new fracture development.

	New fracture	*n*	*X*	Statistical significance	*p*
Mobilization time (hours)	No	105	9.45	3.33	0.75
	Yes	21	9.19	3.54	

**Table 4:** Statistical results evaluating the relationship between the mobilization time (hours) and adjacent or nonadjacent segment fracture formation among new fractured patients.

	Fracture	*n*	*X*	Statistical significance	*p*
Mobilization time (hours)	Adjacent segment	11	9.3	3.93	0.48
	Nonadjacent segment	10	8.60	3.17	

## Data Availability

The data used to support the findings of this study are available from the corresponding author upon request.
